# Effect of Oral Inflammatory Burden on Size and Stage of Oral Tongue Cancer

**DOI:** 10.1111/odi.15227

**Published:** 2024-12-30

**Authors:** Orvokki Saraneva, Jussi Furuholm, Jaana Hagström, Timo Sorsa, Ville Rita, Taina Tervahartiala, Hannamari Välimaa, Hellevi Ruokonen

**Affiliations:** ^1^ Department of Oral and Maxillofacial Diseases University of Helsinki Helsinki Finland; ^2^ Department of Oral and Maxillofacial Surgery University of Helsinki and Helsinki University Hospital Helsinki Finland; ^3^ Department of Pathology University of Helsinki and Helsinki University Hospital Helsinki Finland; ^4^ Department of Oral Pathology and Radiology University of Turku Turku Finland; ^5^ Division of Periodontology, Department of Dental Medicine Karolinska Institute Stockholm Sweden; ^6^ Department of Virology University of Helsinki Helsinki Finland; ^7^ Department of Infectious Diseases, Meilahti Vaccine Research Center MeVac University of Helsinki and Helsinki University Hospital Helsinki Finland

**Keywords:** *Candida*, inflammatory burden, oral cancer size and stage, sex, smoking, squamous cell carcinoma

## Abstract

**Objective:**

Oral tongue squamous cell carcinoma is an aggressive life‐threatening disease, the prognosis of which is affected by tumor stage and size. We retrospectively studied whether oral inflammatory burden and established tongue carcinoma etiological factors exert an impact on tumor size and stage.

**Materials and Methods:**

Medical records of 183 subjects diagnosed with tongue carcinoma at Helsinki University Hospital were investigated. Data on sex, smoking habits, alcohol consumption, and oral inflammatory burden were assessed by the Periodontal Burden Index, Total Dental Index, and Panorama Tomography Index. In addition, *Candida* hyphae in histological samples, and tumor size and stage were recorded and analyzed. History of oral potentially malignant disorders was also investigated.

**Results:**

Increased oral inflammatory burden, male sex, and smoking were associated significantly with larger size and advanced stage of cancer, whereas oral *Candida* hyphae were not associated with size of tongue carcinoma but were associated with female sex.

**Conclusion:**

Male sex, oral inflammatory burden, and smoking were more common in patients with a large and advanced stage of tongue carcinoma. Thus, oral and periodontal infections and their pro‐inflammatory effects may eventually promote carcinoma growth and advance the stage, especially in males.

## 1 Introduction

About 175 Finns are diagnosed yearly with oral tongue squamous cell carcinoma (OTSCC), making it the most common oral cancer type in Finland (*Suomen Syöpärekisteri*, n.d.). OTSCC is an aggressive disease with a poor long‐term prognosis. The 5‐year OTSCC‐specific survival rate in Finland has been reported to range from 47% to 70% (Mäkitie et al. 2007; Mroueh et al. 2017), in stage I being 87%, stage II 73%, stage III 69%, and stage IV 51% (Mäkitie et al. 2007; Mroueh et al. 2017). Prognosis and survival depend on many factors. Tumor size and stage (T) are used to determine optimal treatment despite they having low prognostic value (Mroueh et al. 2017; Seoane et al. 2010).

Some previous studies indicate that sex has an impact on the size and stage of oral cancer lesions (Kruse, Bredell, and Grätz 2011). Males are known to have less frequent dental visits and treatment than females, and their dental self‐care is less meticulous than their female counterparts (Lipsky et al. 2021; Su et al. 2022). Moreover, the dental health of oropharyngeal and tongue cancer patients is poorer than in the general population (Jouhi et al. 2022).

In this retrospective study, we investigated the effect of oral inflammatory burden and established oral cancer risk factors, that is, premalignant lesions oral leukoplakia, oral lichen planus (OLP), and lichenoid reaction (LR) on the size and TNM stage of OTSCC patients.

## 2 Materials and Methods

### 2.1 Study Design

Data were retrospectively gathered from electronic medical records of 183 patients with OTSCC treated or diagnosed in 2016–2017 at the Department of Oral Maxillofacial Diseases, Helsinki University Hospital, Finland. The patients were identified using the International Classification of Diseases, Tenth Revision (ICD‐10) codes C01, C02.1, C02.11, C02.2, and C02.3. The study cohort has been described in our previous work (Saraneva et al. 2023).

Oral inflammatory burden was evaluated by using the Periodontal Inflammatory Burden Index (PIBI), Total Dental Index (TDI), and Panoramic Tomography Index (PTI). PIBI describes the inflammatory load caused by periodontitis and TDI the inflammatory load caused by oral infections. PTI is used to describe the long‐term inflammatory load caused by oral infections (Lahdentausta et al. 2018; Mattila et al. 1989; Ruokonen et al. 2017). These three indices are described in more detail in our earlier study (Saraneva et al. 2023).

Information about demographics, smoking habits, alcohol consumption, tumor size and stage, lymph node involvement, and metastases was collected. Data on history of oral leukoplakia, OLP, and LR, which are all classified as oral potentially malignant disorders (OPMDs), were included from the patient records. The observation of *Candida* hyphae in biopsy specimens was recorded.

Oral mucosal tissue samples, which included OPMD and OTSCC samples, were processed with hematoxylin and eosin and by Periodic acid–Schiff (PAS) staining to visualize *Candida* hyphae using routine diagnostic methods. The World Health Organization diagnostic criteria were used for the determination of LR and OLP (van der Meij, Mast, and van der Waal 2007; van der Meij and van der Waal 2003). Due to the retrospective nature of the study and the lack of precise differential diagnostic information on clinical manifestation, patients with LR and/or OLP were analyzed as one group. For the same reasons, we were unable to acquire clinical information to meet all criteria for *Candida* infection (Coronado‐Castellote and Jiménez‐Soriano 2013), therefore intraepithelial *Candida* hyphae demonstrated by PAS staining was used as the criterion of active candidiasis.

### 2.2 Statistical Analysis

Our research team performed statistical analyses using the IBM SPSS software package for Macintosh (version 28.0, IBM Corp., Armonk, NY, USA). We cross‐tabulated and analyzed categorical variables with Pearson's Chi‐square test or Fisher's exact test, applying the latter if expected values in cell frequencies were below 5. We also conducted pairwise comparisons as post hoc analyses for Pearson's Chi‐square test, using a *Z* test with a Bonferroni correction for multiple comparisons. We used Pearson's product–moment correlation test to assess correlation between continuous variables. After bivariate comparisons, we conducted binomial logistic regression using the variables smoking, presence of Candida, OPMDs, PTI, and sex as covariates. Throughout the study, we considered *p*‐values below 0.05 as statistically significant.

### 2.3 Ethical Considerations

The study protocol was approved by the Helsinki and Uusimaa Hospital District Ethics Committee, Helsinki, Finland (DNRO HUS/996/2018) and conducted in adherence with the Declaration of Helsinki.

## 3 Results

Demographic data of the patients are presented in Table 1. Due to the retrospective nature of the study, panoramic tomography was found only in 155 patients, and clinical dental and periodontal statuses in 58 and 35 patients, respectively. Thus, TDI and PIBI could be calculated only for these groups. PTI was calculated for 155 patients.

**TABLE 1:** Characteristics of study participants.

Characteristics	*N* (%)
Total	183 (100.0)
Sex	
Female	81 (44.3)
Male	102 (55.7)
Age (years)	60.4 ± 13.68 (median 60.5)
Smoking	
Current	56 (30.6)
Former	25 (13.7)
No	52 (28.4)
Record unavailable	50 (27.3)
Cigarette pack‐years^a^	40 (30–41)
Autoimmune disease (*n* = 174)	25 (13.7)
Immunosuppressive medication (*n* = 174)	20 (10.9)
Oral mucosal diseases	
Leukoplakia	49 (26.8)
Lichen planus and/or lichenoid reaction	48 (26.2)
Candida (before OTSCC)	13 (7.1)
Candida (OTSCC)	18 (9.8)
Verrucous leukoplakia	5 (2.7)
Number of teeth (*n* = 159)	25 (21–28)
TDI (*n* = 58)	3 (1–4)
PTI (*n* = 155)	1 (0–3)
PIBI (*n* = 35)	1 (0–20)

*Note:* Data presented as means ± standard deviations or medians (and interquartile ranges) for continuous variables and as frequencies (and percentages) for categorical variables.

Abbreviations: OTSCC, oral tongue squamous cell carcinoma; PIBI, Periodontal Inflammatory Burden Index; PTI, Panoramic Tomography Index; TDI, Total Dental Index.

^a^ Record available for 41 current or former smokers.

Higher oral inflammatory burden values were correlated with larger oral tongue tumor sizes when assessed by TDI, PIBI, and PTI. When stratified by sex, the correlation remained significant in men by TDI and PTI, whereas in women, it was detectable by PIBI (Table 2). Size of OTSCC was significantly associated with male sex (Table 3) and with smoking (Table 4). Binomial logistic regression showed that former smokers (*p* = 0.019) and patients with higher PTI values (*p* = 0.046) had greater odds for larger OTSCC lesions. Records of alcohol use were mostly unavailable and could not therefore be reliably analyzed.

**TABLE 2:** Correlation between oral inflammatory burden variables and tumor characteristics, that is, size of tumor and lymph node involvement.

All, *N* = 183	TDI (*n* = 58)	PIBI (*n* = 35)	PTI (*n* = 155)
Size of tumor (T)	0.315 (0.016)*	0.367 (0.028)*	0.308 (< 0.001)***
Lymph node involvement (N)	0.016 (0.905)	−0.112 (0.516)	0.081 (0.323)
Women, *n* = 81			
Size of tumor (T)	−0.102 (0.634)	0.570 (0.033)*	0.213 (0.489)
Lymph node involvement (N)	−0.213 (0.318)	0.071 (0.810)	0.061 (0.644)
Men, *n* = 102			
Size of tumor (T)	0.425 (0.012)*	0.248 (0.266)	0.330 (0.002)**
Lymph node involvement (N)	0.240 (0.171)	−0.212 (0.344)	0.087 (0.414)

*Note:* Data presented as Pearson's correlation coefficient, *r* (*p* value).

Abbreviations: PIBI, Periodontal Inflammatory Burden Index; PTI, Panoramic Tomography Index; TDI, Total Dental Index.

* Statistically significant (*p* < 0.050).

** Statistically significant (*p* < 0.010).

*** Statistically significant (*p* < 0.001).

**TABLE 3:** Study variables T and N stratified by sex.

Characteristics	Females (*n* = 81)	Males (*n* = 102)	Total (*N* = 183)	*p*
Size of tumor (T), data available	*n* = 76	*n* = 99	*n* = 175	0.020*
1	52 (68.4)	50 (50.5)	100 (58.3)	
2	21 (27.6)	31 (31.3)	52 (29.7)	
3	1 (1.3)	5 (5.1)	6 (3.4)	
4	2 (2.6)	13 (13.1)	15 (8.6)	
Lymph node involvement (N), data available	*n* = 75	*n* = 99	*n* = 174	0.300
0	62 (82.7)	79 (79.8)	141 (81.0)	
1	8 (10.7)	7 (7.1)	15 (8.6)	
2	5 (6.7)	13 (13.1)	18 (10.3)	

*Note:* Data presented as frequencies (and percentages).

* Statistically significant (*p* < 0.050).

**TABLE 4:** Smoking and size of tumor.

Characteristics	Non‐smokers (*n* = 52)	Current smokers (*n* = 25)	Former smokers (*n* = 56)	*p*
Size of tumor (T), data available	*n* = 50	*n* = 25	*n* = 55	0.018*
1	31 (62.0)	14 (56.0)	22 (40.0)	
2	14 (28.0)	11 (44.0)	20 (36.4)	
3	3 (6.0)	0 (0)	2 (3.6)	
4	2 (4.0)	0 (0)	11 (20.0)	
Lymph node involvement (N), data available	*n* = 52	*n* = 24	*n* = 54	0.364
0	43 (82.7)	19 (79.2)	40 (74.1)	
1	6 (11.5)	1 (4.2)	5 (9.3)	
2	3 (5.8)	4 (16.7)	4 (16.7)	

*Note:* Data presented as frequencies (and percentages).

* Statistically significant (*p* < 0.050).

OPDM had been earlier diagnosed in 97/183 patients (53.0%). PAS staining revealed *Candida* hyphae in the OTSCC sample in 18 of 183 patients (9.8%) and in 13 of the 97 OPMD samples (13.4%), which is 7.1% of all 183 patients (Table 1). Oral *Candida* hyphae was not associated significantly with the size of OTSCC (Table 5). History of OLP/LR or leukoplakia diagnosis was not associated with tumor size (Table 6) or with the presence of oral *Candida* hyphae.

**TABLE 5:** Study variables stratified by presence of *Candida* by histopathologic examination and tumor size.

Characteristics	*Candida* pos. (*n* = 18)	*Candida* neg. (*n* = 145)	*Candida* pos. only before cancer diagnosis (*n* = 13)	*p*
Size of tumor (T), data available	*n* = 17	*n* = 145	*n* = 13	0.059
1	13 (76.5)	80 (55.2)	9 (69.2)	
2	2 (11.8)	47 (32.4)	3 (23.1)	
3	2 (11.8)	3 (2.1)	1 (7.7)	
4	0	15 (10.3)	0	
Lymph node involvement (N), data available	*n* = 16	*n* = 145	*n* = 13	
0	14 (87.6)	114 (78.6)	13 (100.0)	0.639
1	1 (6.2)	14 (9.7)	0	
2	1 (6.2)	17 (11.7)	0	

*Note:* Data presented as frequencies (and percentages).

**TABLE 6:** Study variables stratified by presence of oral mucosal diseases.

Characteristic	Leukoplakia, no OLP/LR (*n* = 38)	OLP/LR, no leukoplakia (*n* = 37)	Leukoplakia and OLP/LR (*n* = 11)	Neither leukoplakia nor OLP/LR (*n* = 97)	*p*
Size of tumor (T), data available	*n* = 38	*n* = 35	*n* = 11	*n* = 91	0.148
1	23 (60.5)	22 (62.9)	10 (90.9)	47 (51.6)	
2	12 (31.6)	12 (34.3)	1 (9.1)	27 (29.7)	
3	1 (2.6)	1 (2.9)	0	4 (4.4)	
4	2 (5.3)	0	0	13 (14.3)	
Lymph node involvement (N), data available	*n* = 37	*n* = 35	*n* = 11	*n* = 92	0.216
0	31 (83.8)	32 (91.4)	8 (80.0)	70 (76.1)	
1	2 (5.4)	0	2 (20.0)	11 (12.0)	
2	4 (10.8)	3 (8.6)	0	11 (12.0)	

*Note:* Data presented as frequencies and percentages.

Abbreviation: OLP/LR, oral lichen planus and/or lichenoid reaction.

Of all patients (*n* = 183), there was lymph node involvement in 33 (18.0%) patients. Lymph node involvement was not associated with any predictor variables (Tables 2, 3, 4, 5, 6).

## 4 Discussion

This is a retrospective study of 183 tongue squamous cell carcinoma patients from Helsinki University Hospital (HUS) area and based on our previous study population reported in the article Saraneva et al. 2023 (ref). HUS is the largest university hospital area in Finland with a population of 2.2 million inhabitants. Even though the sample size is relatively small, it is representative of this population.

The most important finding in our study was that increased oral inflammatory burden was significantly associated with larger size and advanced stage of the OTSCC lesion. However, due to the retrospective nature of the study, dental and periodontal statuses were not available for all patients. This emphasizes the importance of a dentist being part of the treatment team.

In earlier studies, oral inflammation has been linked to oral cancer invasion. Oral pathogenic bacteria can contribute to oral carcinogenesis via a number of potential mechanisms. Of the periodontal pathogens, *T*. *denticola* , *P*. *gingivalis* , and *F*. *nucleatum* have been shown to promote oral carcinogenesis via OSCC cell migration, invasion, and to contribute to oral aggressiveness (Kamarajan et al. 2020). *P*. *gingivalis* can also promote migration and invasion of OSCC cells by triggering epithelial to mesenchymal transition‐like changes and acquisition of stemness properties (Ha et al. 2015). These events can contribute to larger size and stage of OTSCC lesions. However, in this study, periodontal pathogens were not investigated in periodontal samples due to the retrospective nature of the study, which is a limitation.

Male sex was significantly associated with size of OTSCC. Some earlier studies have found no difference between the sexes in the size and stage of oral cancer (Kruse, Bredell, and Grätz 2011), while others have reported that men aged 45–64 years have an increased prevalence of advanced stage oral cancer (Lins et al. 2019) and women being diagnosed at an older age and earlier tumor stage (Lee et al. 2021). Previous studies have also reported that males have more irregular dental visits and poorer oral self‐care than females, and they seek treatment less eagerly (Lipsky et al. 2021; Su et al. 2022). A delay in diagnosis could contribute to larger tumor size.

Hormonal influences on oral cancer have been a subject of interest. Hormones, particularly estrogen, progesterone, and testosterone, play roles in various physiological processes, including cell growth and differentiation. In oral carcinogenesis, studies have shown that hormone receptors, like estrogen receptors, could be regarded as a biological predisposition factor for OSCC (Grimm et al. 2016). Estrogen receptors have a potentially important role in cell survival in OSCC, and their inhibition prevents tumor cell invasion and possibly metastasis (Ishida et al. 2007). Estrogen was found to stimulate OSCC invasion in vitro (Egloff et al. 2009; Ishida et al. 2007). On the other hand, estrogen has been suggested to have potential protective effects against oral cancer due to its role in maintaining the integrity of mucosal tissues (Olsson, Bladström, and Ingvar 2003). Oral cancer develops at an older age in females than in males, and it can be assumed that women are protected against oral cancer in their reproductive period (Suba 2007). The larger tumor size in males might be due to hormonal differences, but this hypothesis remains to be clarified in future studies.

In previous studies, contradictory results on lymph node involvement in OTSCC have been reported. One study noted similar findings between the sexes (Lee et al. 2021), whereas another study found cervical lymph node metastases to be slightly more common in female patients (Kruse, Bredell, and Grätz 2011). In our study, no significant differences emerged in lymph node involvement between the sexes.

We found an association between smoking and the size of OTSCC: larger OTSCCs were most common in former smokers (Table 4). In an earlier study, the concomitant use of alcohol and smoking has been reported to increase the prevalence of advanced stage cancer (Bezerra et al. 2018).

Oral *Candida* hyphae were not significantly associated with the size of OTSCC or with oral potentially malignant lesions. No *Candida* was found even in grade IV OTSCC samples, although *Candida* species are believed to have a role in oral carcinogenesis (Alnuaimi et al. 2016; Bombeccari, Giannì, and Spadari 2017; Krogh et al. 1987). In previous studies, OSCC patients have been found to be more often colonized with *Candida* species than controls (Alnuaimi et al. 2015). *Candida* isolates from OTSCC patients are reported to produce more virulence factors than isolates from non‐OTSCC patients (Castillo, Sotomayor, and Azcurra 2018). *Candida* from OSCC patients are more prone to form biofilms, to produce more carcinogenic acetaldehyde, and to have higher metabolic activity than biofilms from non‐oral cancer (Alnuaimi et al. 2016). *C. albicans* has been shown to produce carcinogens, including nitrosamines, which could trigger carcinomatous changes (Krogh et al. 1987; Krogh 1990). *Candida* proteases also activate latent proMMP‐8 and promote tissue destruction (Pärnänen et al. 2020). *Candida* species may produce potentially carcinogenic amounts of acetaldehyde, especially in combination with smoking or alcohol consumption (Alnuaimi et al. 2016).

Although *Candida* is believed to have a role in oral carcinogenesis, in our study, *Candida* was not associated with larger cancer lesions. This can be explained by the fact that *Candida* in our study was more common in females, while males had larger cancer lesions. Interestingly, we did not detect any *Candida* in grade IV OTSCC lesions. Whether *Candida* also plays a role in large size and advanced stage of oral cancer lesions to reduce cancer growth remains to be answered in future studies.

The retrospective nature of this study set limitations on the data available, especially regarding alcohol consumption, which would have been relevant variable to investigate. We were also unable to make a more precise classification of OPMDs. Any previous diagnosis of oral leukoplakia, OLP, and LR, that is, oral potentially premalignant disorders (OPMD), were ascertained from histopathological reports of 183 oral tongue carcinoma patients. The data does not include patients with premalignant oral disorder without malignant transformation, which is a shortcoming of the study. It is important to note that not all OSCCs are preceded by OPMD. In addition, we recognized the small sample size as a limitation of the study.

In conclusion, our results suggest that oral and periodontal infections and their pro‐inflammatory effects may eventually promote carcinoma growth and advance the stage, especially in males.

## Author Contributions

**Orvokki Saraneva:** conceptualization, methodology, investigation, writing – original draft, writing – review and editing. **Jussi Furuholm:** conceptualization, methodology, investigation, statistical analysis, writing – review and editing. **Jaana Hagström:** conceptualization, methodology, writing – review and editing. **Timo Sorsa:** conceptualization, writing – review and editing. **Ville Rita:** investigation, writing – review and editing. **Taina Tervahartiala:** conceptualization, writing – review and editing. **Hannamari Välimaa:** conceptualization, methodology, resources, supervision, writing – review and editing. **Hellevi Ruokonen:** conceptualization, methodology, resources, project administration, supervision, writing – review and editing.

## Ethics Statement

The study protocol was approved by the Helsinki and Uusimaa Hospital District Ethics Committee, Helsinki, Finland (DNRO HUS/996/2018) and conducted in adherence with the Declaration of Helsinki.

## Consent

This is a retrospective study, and therefore, no written consent was required.

## Conflicts of Interest

The authors declare no conflicts of interest.

## Data Availability

The data that support the findings of this study are available on request from the corresponding author. The data are not publicly available due to privacy or ethical restrictions.
